# Affect school and script analysis versus basic body awareness therapy in the treatment of psychological symptoms in patients with diabetes and high HbA1c concentrations: two study protocols for two randomized controlled trials

**DOI:** 10.1186/s13063-016-1347-8

**Published:** 2016-04-27

**Authors:** Eva O. Melin, Ralph Svensson, Sven-Åke Gustavsson, Agneta Winberg, Ewa Denward-Olah, Mona Landin-Olsson, Hans O. Thulesius

**Affiliations:** Department of Clinical Sciences, Endocrinology and Diabetes, Lund University, Lund, Sweden; Department of Research and Development, Region Kronoberg, Box 1223, SE-351 12 Växjö, Sweden; Primary Care, Region Kronoberg, Växjö, Sweden; Department of Psychology, Linnaeus University, Växjö, Sweden; Department of Psychiatry, Region Kronoberg, Växjö, Sweden; Department of Endocrinology, Lund University Hospital, Lund, Sweden; Department of Clinical Sciences, Family Medicine, Lund University, Malmoe, Sweden

**Keywords:** Alexithymia, Anxiety, Cortisol, Depression, Diabetes mellitus, HbA1c, Mind-body therapy, Randomized controlled trial, Psycho-education

## Abstract

**Background:**

Depression is linked with alexithymia, anxiety, high HbA1c concentrations, disturbances of cortisol secretion, increased prevalence of diabetes complications and all-cause mortality. The psycho-educational method ‘affect school with script analysis’ and the mind-body therapy ‘basic body awareness treatment’ will be trialled in patients with diabetes, high HbA1c concentrations and psychological symptoms. The primary outcome measure is change in symptoms of depression. Secondary outcome measures are changes in HbA1c concentrations, midnight salivary cortisol concentration, symptoms of alexithymia, anxiety, self-image measures, use of antidepressants, incidence of diabetes complications and mortality.

**Methods:**

Two studies will be performed. Study I is an open-labeled parallel-group study with a two-arm randomized controlled trial design. Patients are randomized to either affect school with script analysis or to basic body awareness treatment. According to power calculations, 64 persons are required in each intervention arm at the last follow-up session. Patients with type 1 or type 2 diabetes were recruited from one hospital diabetes outpatient clinic in 2009. The trial will be completed in 2016. Study II is a multicentre open-labeled parallel-group three-arm randomized controlled trial. Patients will be randomized to affect school with script analysis, to basic body awareness treatment, or to treatment as usual. Power calculations show that 70 persons are required in each arm at the last follow-up session. Patients with type 2 diabetes will be recruited from primary care. This study will start in 2016 and finish in 2023. For both studies, the inclusion criteria are: HbA1c concentration ≥62.5 mmol/mol; depression, alexithymia, anxiety or a negative self-image; age 18–59 years; and diabetes duration ≥1 year. The exclusion criteria are pregnancy, severe comorbidities, cognitive deficiencies or inadequate Swedish. Depression, anxiety, alexithymia and self-image are assessed using self-report instruments. HbA1c concentration, midnight salivary cortisol concentration, blood pressure, serum lipid concentrations and anthropometrics are measured. Data are collected from computerized medical records and the Swedish national diabetes and causes of death registers.

**Discussion:**

Whether the “affect school with script analysis” will reduce psychological symptoms, increase emotional awareness and improve diabetes related factors will be tried, and compared to “basic body awareness treatment” and treatment as usual.

**Trial registration:**

ClinicalTrials.gov: NCT01714986

## Background

Diabetes is a progressive and demanding chronic disease, which might cause secondary distress requiring emotional adaptation [1]. An increased prevalence of depression is found in patients with diabetes [2, 3]. The comorbidity is linked with inadequate glycemic control [4], disturbances in cortisol secretion [3, 5], increased prevalence of diabetes complications [6, 7] and all-cause mortality [7].

Affects are the innate, unconscious and strictly biological portions of emotions [8–12]. Deficits in the awareness of affects result in abnormal physiological reactions and impaired capacity for self-care and self-regulation [13]. Alexithymia includes difficulties in identifying and describing feelings, as well as low capacity for introspection and reflection [13, 14]. Alexithymia has been demonstrated as a risk factor of depression but the number and prevalence of alexithymic features might also increase during depressive episodes [15]. Type 1 diabetes [16], inadequate glycemic control of diabetes [17], reflexive emotional eating and obesity [18–20] and increased cardiovascular mortality [21] have been linked to alexithymia. Anxiety has been associated with depression [4] and inadequate glycemic control [22].

A negative self-image, which includes self-hate, self-ignorance or self-blame, is often present in persons with eating disorders [23], which, in turn, are associated with inadequate glycemic control [24].

High concentrations of HbA1c [25, 26] and disturbances in cortisol secretion [27, 28] are linked to complications of diabetes.

The psycho-educational method ‘affect school with script analysis’ (ASSA) was constructed by Bergdahl and Armelius [29, 30], inspired by Silvan S. Tomkins’ affect theory [8–11] and its interpretation by Nathanson [12] and Monsen and Monsen [31]. This method has previously been shown to decrease alexithymia [32] and stress symptoms [30].

‘Basic body awareness therapy’ (BBAT) is a physiotherapeutic mind-body therapy [33], where increased body awareness and increased integration between body and mind are key elements, as in other mind-body therapies such as Tai Chi, yoga, meditation and mindfulness-based therapies. In Sweden, BBAT is used in both psychiatric and primary care settings for treating psychiatric and somatic health issues [34–36].

### Objective and research questions

#### Studies I and II

The main hypothesis in these two studies is that an increased emotional awareness and verbal expressiveness might have beneficial health implications, such as lessening of depressive symptoms. The primary outcome measure is change in symptoms of depression. Secondary outcome measures are changes in symptoms of alexithymia, anxiety, self-image measures, HbA1 concentrations, midnight salivary cortisol concentration, use of antidepressants and incidence of diabetes complications and mortality.

#### Study I

The main aim is to compare the effects of ASSA and BBAT in patients with type 1 or type 2 diabetes, psychological symptoms and inadequate glycemic control.

#### Study II

The main aim is to compare ASSA, BBAT with only treatment as usual in patients with type 2 diabetes, psychological symptoms and inadequate glycemic control.

## Methods/design

### Study design

These studies were registered with ClinicalTrials.gov on 24 October 2012 (NCT01714986). The final report will follow the CONSORT extension for non-pharmaceutical interventions.

Intervention with ASSA and BBAT will be tried in two studies with different study designs for patients with type 1 or type 2 diabetes, psychological symptoms and inadequate glycemic control in Region Kronoberg, Sweden. Results from the two studies will be presented separately but will also be compared.

#### Study I

This is an open-labeled parallel-group study with a two-arm randomized controlled trial design, which was initiated in 2009 and will be complicated in 2016 for patients with type 1 or type 2 diabetes recruited from one hospital diabetes outpatient clinic.

#### Study II

This is a multicentre open-labeled parallel-group study with a three-arm randomized controlled trial design, which will start in 2016 and will be completed in 2023 for patients with type 2 diabetes recruited from primary care units.

### Participants and procedure

#### Inclusion criteria for baseline analysis (Fig. ***1***)

**Fig. 1:**
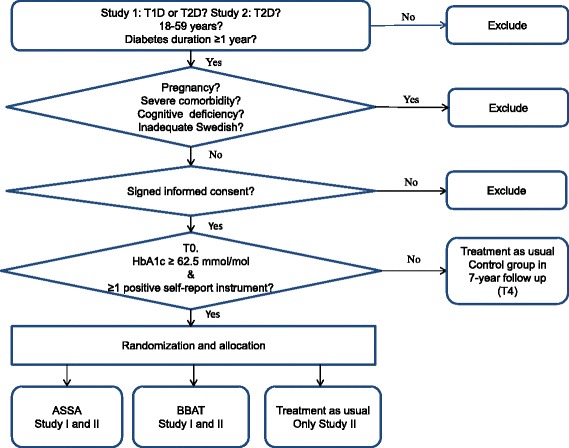
Description of criteria for randomization and allocation to ASSA (Studies I and II), BBAT (Studies I and II) and treatment as usual (Study II)

These are outlined in Fig. 1. For Study I, participants must have either type 1 or type 2 diabetes. For Study II, participants must have type 2 diabetes. For both studies, participants must be aged 18–59 years, have had diabetes for at least 1 year and must provide signed informed consent.

#### Exclusion criteria

These are outlined in Fig. 1. For both studies, exclusion criteria are:

- Pregnancy
- Severe comorbidities, if it is anticipated that participation would be difficult or unsafe for the patient (end-stage renal disease, cancer, hepatic failure, visual impairment to such a degree that reading questionnaires would be impossible, impaired hearing to such a degree that participation in discussions during the group sessions would not be possible, psychotic disorders, bipolar disorder, suicide ideation, severe personality disorder, or severe substance abuse)
- Cognitive deficiencies (mental retardation, stroke or dementia)
- Inadequate knowledge of Swedish.

#### Intervention criteria

For both studies, these are either a HbA1c concentration of at least 62.5 mmol/mol or at least one of: self-reported depression, anxiety, alexithymia or a negative self-image (Fig. 1).

#### Recruitment

In both studies, patients will be recruited at regular bi-annual visits for diabetes control. Nurses specializing in diabetes will send detailed information about the study together with calls for diabetes controls to patients without known criteria for exclusion. At the diabetes control visit, the information will be repeated, and re-evaluation for prevalent exclusion criteria will be performed. The recruitment period will last for 9 months at each unit. Baseline data and measurements (T0) will be collected after signed informed consent has been obtained. The patients are informed that they can withdraw from the study at any time without negative consequences.

#### Randomization and allocation

The randomization and allocation of the coded patients will be performed by an independent statistician with the purpose of having as equal distribution as possible of sex and age in each intervention arm.

##### Study I

**Fig. 2:**
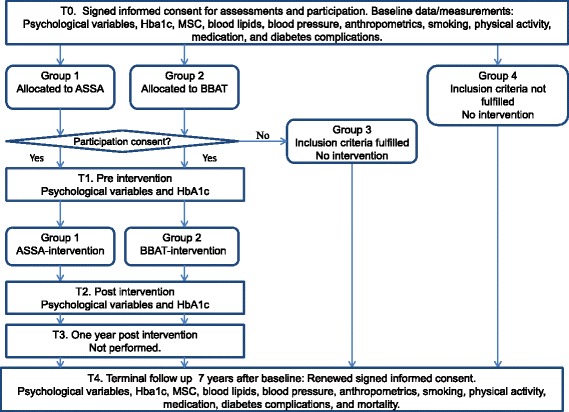
Study flow chart for Study I

This is outlined in Fig. 2. Patients who fulfilled the criteria were randomized into two intervention arms, ASSA (group 1) or BBAT (group 2). After allocation, the ASSA and BBAT instructors offered renewed oral information and patients made their final decisions regarding participation. Group 3 consists of patients who fulfilled the criteria but refused participation after allocation, and group 4 consists of patients who did not fulfil the criteria. Groups 3 and 4 will be followed as comparison groups.

##### Study II

**Fig. 3:**
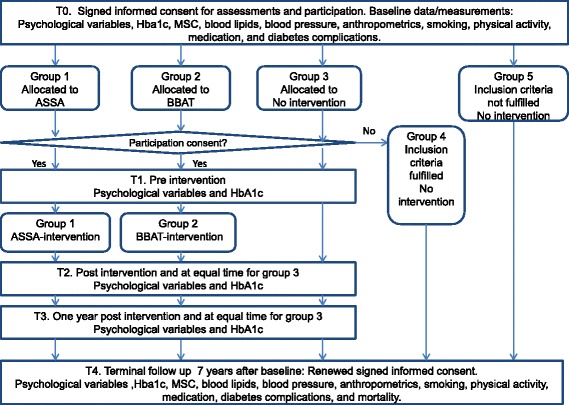
Study flow chart for Study II

This is outlined in Fig. 3. Patients fulfilling the intervention criteria will be randomized into three arms: ASSA (group 1), BBAT (group 2) or no intervention (treatment as usual; group 3). Group 4 will consist of patients who fulfil the criteria but refuse participation after allocation. Group 5 will consist of patients who do not fulfil the criteria.

#### Schedule for assessments

##### Study I

This is shown in Fig. 2. Assessments were performed for participants in ASSA and BBAT at baseline (T0), prior to intervention (T1) and after termination of the intervention (T2) and will be performed 7 years after T0 (T4). For nonparticipants (Groups 3 and 4), assessments were performed at T0 and will be repeated at T4.

##### Study II

Assessments will be performed for Groups 1, 2 and 3 at T0, T1 and T2; 1 year after T2 (T3) and at T4. For Groups 4 and 5, assessments will be performed at T0 and T4.

#### Assessments, measurements and data collection at T0 and T4

##### Studies I and II

Assessments will be performed using self-report instruments; HbA1c concentration, midnight salivary cortisol concentration, blood lipids, blood pressure, and anthropometrics will be measured (Figs. 2 and 3). Data will be retrieved from computerized medical records and from the Swedish national diabetes register. At T4, mortality data will be collected from the Swedish causes of death register.

#### Assessments and measurements at T1, T2 and T3

For Study I, assessments will be made using self-report instruments and HbA1c concentrations will be measured at T1 and T2 (Fig. 2).

For Study II, assessments will be performed with self-report instruments and HbA1c will be measured at T1, T2 and T3 (Fig. 3).

### Additional data collection concerning medication between measuring points

Data will be collected regarding changes in medication that occur between measuring points. The use of antidepressants will be monitored. There will be three categories: (1) no antidepressants; (2) use of a single antidepressant drug; (3) combinations of antidepressant drugs. Amounts of stops and starts will be registered as well as the total time for each category. Type of antidepressants according to official classification will be registered. The use of insulin will be categorized as: (1) no insulin; (2) single or multiple daily injections; (3) continuous subcutaneous insulin infusion. The duration of the period of each category will be monitored. All non-insulin antidiabetic drugs will be registered according to their official classification and the time that participants have used each drug will be monitored.

### Intention to treat analysis

#### Studies I and II

Differences at T0 between participants and nonparticipants fulfilling criteria will be analyzed regarding age, sex, self-reported depression and anxiety, alexithymia, self-image, clinical psychiatric diagnoses, smoking, physical inactivity, metabolic variables, medication and sick-leave status and will be thoroughly described. Levels of HbA1c will be determined for all patients who participate in the interventions, but decline to complete the questionnaires at T2, T3 (Study II only) or T4. At T4, all patients who participated at T0 will be asked for oral and written consent to participate in all the assessments, measurements and data collection that are included at T4. If full assessments are not possible we will collect data regarding HbA1c concentrations, mortality, diabetes complications and use of antidepressants.

### Written and oral informed consent

Written and oral informed consent will be received from each participant in both studies at T0 and at T4, and oral consent after allocation to intervention (Figs. 2 and 3).

The studies were approved by the Regional Ethical Review Board of Linköping University, Linköping (registration no. M120-07, T83-08).

### Self-report instruments

#### Studies I and II

The Hospital Anxiety and Depression Scale will be used at all measuring points to assess self-reported anxiety and depression [4, 5, 32, 37–39]. The cut-off level for each subscale recommended by the constructors of this scale (≥8 points) will be used for inclusion in the intervention [38]. In a study that compared the Swedish version of the Hospital Anxiety and Depression Scale with a structured clinical interview for DSM-III criteria, the optimal cut-off level for screening was ≥5 points for both subscales [39].

The 20-item Toronto Alexithymia Scale will be used at all measuring points to assess alexithymia [4, 19, 32, 37, 40–42]. This scale consists of 20 statements rated from 1 to 5, and is based on three subscales: ‘difficulty identifying feelings’ (seven statements), ‘difficulty describing feelings’ (five statements) and ‘externally oriented feelings’ (eight statements).The recommended cut-off level (≥61 points) will be used for inclusion in the intervention [41, 42].

The affinity dimension of the Structural Analysis of Social Behavior assessment tool will be used to assess self-image [23, 35, 43]. This test consists of 36 statements with response options on a scale between 0 and 100; the results are summarized into eight clusters. High scores for the clusters ‘blaming self’, ‘hating self’ and ‘ignoring self’; and low scores for the clusters ‘accepting self’, ‘loving self’ and ‘nourishing self’ form a negative self-image. A negative self-image is defined as results below 284 points. The Structural Analysis of Social Behavior test will be used at T0 and at T4.

### Measurements

#### Study I and II

According to the Swedish national guidelines for diabetes management in 2009, the recommended goal for treatment was for HbA1c concentrations of less than 52 mmol/mol. However, since goals should be individualized, a higher cut-off value was chosen in this study: venous HbA1c concentration (IFCC units) ≥62.5 mmol/mol, (Mono-S ≥7 %, NGSP ≥7.87 %) [44]. Analyses will be performed with high pressure liquid chromatography, (HPLC - variant II, Turbo analyzer, Bio-Rad®, Hercules, CA, USA) [45].

Serum lipids will be analyzed using the enzymatic colour test (Olympus AU®, Tokyo, Japan). Blood lipids and HbA1c will be analyzed at the Department of Clinical Chemistry, Växjö Central Hospital.

Blood pressure, waist circumference, weight and length will be measured by a nurse, according to standard procedures. Abdominal obesity is defined for men as waist circumference ≥1.02 m. and for women as waist circumference ≥0.88 m [4, 5]. General obesity is defined as body mass index ≥30 kg/m^2^ for both sexes [4].

Midnight salivary samples will be collected between 23.30 and 00.30 hours using a Salivette sampling device [5, 46–49] (Salivette®, Sarstedt, Nümbrecht, Germany). Assays will be performed at the Department of Clinical Chemistry, Lund University Hospital, Lund, using Roche Cobas Cortisolassay®, a competitive electrochemiluminescence immunoassay [5, 46–49], using an Elecsys 2010 immunoanalyzer system (Roche Diagnostics, Mannheim, Germany). Patients will receive written and oral instructions before sampling [5].

### Data collection

For both studies, data regarding patients’ diagnoses, diabetes complications, life style factors, medication, psychotherapy, diabetes-related medication and antidepressants and sick leave will be collected from the Swedish national diabetes register, from medical records from the departments of internal medicine, ophthalmology and psychiatry, and from primary care centres. Data from all hospital clinics, the psychiatry department and all primary care centres (including psychotherapy obtained in primary care) are available as they are connected to the same computer system (Cambio Cosmic ®). Mortality data will be collected from the Swedish causes of death register.

### Definitions of diabetes complications and severe hypoglycemia episodes

Foot complications are defined as neuropathy, angiopathy, previous or current diabetes foot ulcer, foot infection, foot deformity, arthropathy, or amputation of the lower limb [4]. Diabetes retinopathy is defined as nonproliferative or proliferative retinopathy with microangiopathy changes, as viewed by fundus photography through a dilated pupil [4]. Macrovascular complications are defined as ischemic heart disease (angina pectoris, previous myocardial infarction, percutaneous transluminal coronary angioplasty or coronary artery bypass graft surgery); stroke or transient ischemic attack [4]. End-stage renal disease is defined as a state where dialysis or a renal transplant is necessary [50]. A severe hypoglycemic episode is defined as one in which help is needed from another person owing to hypoglycemia; episodes occurring during the previous 6 months will be registered [4, 5].

### Definitions of smoking and physical inactivity

Patients are defined as smokers if they have smoked any amount of tobacco during the last year [4, 5]. In the Swedish national diabetes register, physical activity is categorized into four groups: moderate activities for ≥30 minutes, less than once a week, once a twice a week, 3–5 times/week, or daily. Patients are defined as physically inactive if they perform moderate activities, such as 30 minutes of walking, less than once a week [4, 5].

### Affect school and script analysis – a psycho-educational method

#### Studies I and II

The intervention comprises eight weekly sessions of a group with five to seven participants (‘affect school’) with two instructors followed by ten weekly individual sessions (‘script analysis’). Two psychotherapists and one physician are recruited as instructors for both studies. They were trained together to become instructors by the constructors of the ASSA method, and have been instructors together in several ASSA groups. The same two instructors participate in all eight group sessions, which render three possible combinations: physician and psychotherapist I; physician and psychotherapist II; and psychotherapist I and psychotherapist II. The patients could be considered clustered within the pair of instructors. Patients will also be clustered by the set of five to seven participants included in each group. As depression is the main outcome measure we will test associations between depression symptoms, instructors and groups. Only one room will be used for all group sessions in each study.

At each affect school session a specific information handout is distributed, containing affect and script theory and the topics for the affect discussion. The scheme for the affect school sessions is presented in Table 1. During the first part of each affect school session the participants are instructed on general affect theory, and specific theory regarding the particular innate affects that are the topics for the session, followed by script theory. During the second part, the affect discussion, participants are encouraged to tell single-event autobiographical memory narratives; this exercise is considered important in psychotherapy [51]. The goal of the affect school is to achieve increased emotional awareness and verbal expressiveness. The affect theory presented during the affect school group sessions is essential for the script analysis, and we consider that participation in at least five group sessions is necessary to obtain an understanding of the theory. Persons with lower participation rate will not be offered script analysis. The total participation rate in ASSA will be counted.

**Table 1:** General programme for the eight affect school sessions

*Affect theory*	
General affect theory is presented at all sessions. Specific affect theory for innate affects is presented according to the following program for the eight sessions.	
1) Joy	
2) Fear	
3) Interest and surprise	
4) Shame	
5) Anger	
6) Distaste and dissmell	
7) Distress	
8) Pain	
*Script theory presented at each session*	
1) Affects and experiences together form the individual scripts.	
2) How we act in different situations and how we interpret experiences depending on our scripts.	
3) Scripts are formed by family rules and common cultural rules for how affects should be handled.	
4) Intensity and expressions of emotion are controlled by scripts.	
5) Affects can be completely suppressed and thereby unconscious.	
Break for coffee or tea	
*Affect discussion*	
Main topics for the affect discussions follow the programme for the eight sessions. Questions used in the affect discussions are:	
1) Tell of a situation when you felt…	
2) How do you know that you feel…?	
3) Do you feel it in a particular place in the body?	
4) Does it happen often?	
5) How do you know that someone else is…?	
6) Can you understand and accept another person’s…?	
7) How does… influence your personal relationships?	

During the individual sessions (script analysis), the patient is encouraged to focus on one or two affects that he or she finds particularly difficult to handle, and the patient’s scripts for handling these affects are discussed. These sessions are performed by psychotherapists or social counsellors.

Here follows a short description of the affect theory that will be presented during the group sessions. A more complete description is presented elsewhere [37]. Essential in the affect school are the five concepts of affects, feelings, emotions, mood and scripts. Affects are the innate, unconscious and strictly biological portions of emotions. Each affect has a specific programme involving face mimicry, body gestures, voice and autonomous nervous and hormone system physiology. The innate affects are enjoyment-joy, interest-excitement, surprise-startle, fear-terror, anger-rage, distress-anguish, shame-humiliation, distaste and dissmell. Pain has features of both an affect and a drive. Affects are important messengers to the self. The primary functions of the innate affects are to regulate drives, such as hunger and sexuality. The secondary functions are to regulate other affects. *Feelings* are the conscious portions of emotions. *Emotions* reflect biography. A triggered affect evokes memories of earlier situations and relationships where this affect has been triggered before and, in addition, other affects triggered in the earlier situations will be triggered again in the present situation. An emotion lasts as long as memory continues to trigger the involved affects. *Mood* is defined as a persistent state of emotion. Anxiety and depression are subsequently mood disorders. *Scripts* are learned patterns to handle emotions.

### Basic body awareness therapy – a physiotherapeutic mind-body therapy

#### Studies I and II

This comprises ten weekly sessions for a group of five to seven participants with one instructor responsible for all sessions. Three physiotherapists, with special BBAT education and clinical experience of being BBAT instructors are recruited for both group and individual sessions in both studies. Before intervention, the BBAT instructors meet and synchronize how they will perform the interventions. The patients are clustered by BBAT instructors. Patients will also be clustered by the set of five to seven participants included in each group. We will test associations between depression symptoms, instructors and group. Only one room will be used for all group sessions in each study. Breathing, grounding, stability in the central line, centring, flexibility and flow are features systematically trained at each session. Patients are encouraged to observe and accept the sensations and emotions that awaken during the treatment situations. Patients systematically train to discern and differentiate between thoughts, emotions and body sensations. Continuous training and repetition are fundamental parts of BBAT [36].

Patients are offered five individual sessions where they focus on their own particular difficulties.

To balance the two intervention methods patients participating in fewer than five group sessions will not be offered any individual sessions. The total participation rate will be counted.

Enhanced body awareness and a greater unity between body and self are important goals [33]. Body awareness is the subjective, phenomenological aspect of proprioception and interoception that enters conscious awareness, and is modifiable by mental processes including attention, interpretation, appraisal, beliefs, memories, conditioning, attitudes and affects [33]. Important in BBAT is breathing, which is regarded as a central connector between body and mind. Improved body and mind integration is achieved by noticing, discerning, and differentiating thoughts, emotions and body sensations [33].

### Measures to enhance participation

In both studies, participants are offered ‘preventive sick leave’ for the time spent in group or individual sessions by the Swedish Social Insurance Agency, as a measure to enhance participation.

### Discontinuation of intervention with ASSA and BBAT

In both studies, if anything should happen to patients during the intervention so that they fulfil the exclusion criteria, they will be advised to withdraw. In case of psychiatric complications as a result of the interventions, patients will be offered personal counselling or referral to a psychiatric unit.

### Treatment as usual

In both studies, there will be no restriction on the use of treatment as usual, such as psychotherapeutic or physiotherapeutic treatment, antidepressant medication or diabetes-related treatment.

### Primary and secondary outcome measures

In both studies, the primary outcome measure is the change in depression symptoms. Secondary outcome measures are changes in alexithymia and anxiety symptoms and self-image measures; changes in HbA1c concentration and midnight salivary cortisol concentration; use of antidepressants, and incidence of diabetes complications and mortality.

### Power calculations for sample size

The standard deviation was 2.8 for the individual changes of Hospital Anxiety and Depression Scale – depression subscale after our intervention with ASSA for patients with chronic benign pain (calculated from raw data) [32].

#### Study I

If we assume that the standard deviation for the individual changes in Hospital Anxiety and Depression Scale – depression subscale in this study is 2.8, a change of 1.4 points represents half the standard deviation, which is considered indicative of clinically relevant changes in subjective symptoms [52]. Calculating a statistical power of 80 %, and assuming significance at the 0.05 level, 64 persons in each intervention arm are required at follow-up in a two-arm randomized controlled trial. Unfortunately we only recruited around 21 persons in each arm in Study I, rendering a power of 37 %.

#### Study II

The power calculation is based on an analysis of variance (ANOVA), where two groups have the same means and one differs with 1.4 points, using the same standard deviation (2.8) as in Study I. Calculating a statistical power of 80 %, calculated using an ANOVA, 60 persons are required. However, using the Kruskal–Wallis test reveals that it is necessary to increase this by 15 %; thus, 70 persons are required in each arm in a three-arm randomized controlled trial.

#### Studies I and II

In the previously mentioned intervention with ASSA, approximately 10 % of participants did not answer the self-report instruments after the intervention [32]. If we assume the same answering rate, 10 % more patients have to be included in each arm.

### Statistical analysis

#### Study I

The Wilcoxon signed rank test and the Kruskal–Wallis test will be used for changes in non-normally distributed variables (for psychological symptoms measured using the self-report instruments and for midnight salivary cortisol concentration).

#### Study II

The Kruskal–Wallis test will be used for changes in non-normally distributed variables (psychological symptoms and midnight salivary cortisol concentration).

#### Studies I and II

A paired-samples *t* test will be used for continuous and normally distributed variables (HbA1c concentration). The associations between outcome variables and baseline characteristics will be modelled using linear regression analysis (HbA1c concentration, midnight salivary cortisol concentration, psychological symptoms); logistic regression analysis will be used for categorical outcomes (incidence of diabetes complications and use of antidepressants).

Cox proportional-hazards regression will be used to analyze effects on mortality. Missing values of the self-report instruments will be imputed using multinomial logistic regression on the other variables in the same subgroup [53]. Imputation will be performed for the depression and anxiety subscales of the Hospital Anxiety and Depression Scale if there are at most two missing items for each subscale; for the 20-item Toronto Alexithymia Scale if there is at most one missing item for each subscale (‘difficulty identifying feelings’, ‘difficulty describing feelings’ and ‘externally oriented feelings’) and for the Structural Analysis of Social Behavior tool if there is at most one missing item for each of the eight clusters. Statistical significance will be considered for *P* < 0.05.

## Discussion

Diabetes is a progressive and demanding chronic disease, which requires emotional adaptation [1]. Previous research has shown that alexithymia is a risk factor for depression [15], which is deleterious for patients with diabetes [4–7]. Also, a deficit in the awareness of affects results in abnormal physiological reactions and impaired capacity for self-care [13]. An advanced capacity for self-care is necessary in diabetes.

For this reason, we were interested in finding interventions focusing on increased emotional awareness and verbal expressiveness. We have chosen to test the ASSA [30, 32, 37] against BBAT [34–36] in two studies. In Study I, we perform a two-arm randomized controlled trial with patients with type 1 or type 2 diabetes from one hospital diabetes outpatient clinic. In Study II, ASSA, BBAT and ‘treatment as usual’ will be compared in a three-arm randomized controlled trial for patients with type 2 diabetes from primary care units.

We expect the ASSA method to be more efficient than BBAT in reducing depressive symptoms. In the ASSA intervention, patients are systematically trained to identify and describe feelings, and are also presented with advanced affect theory. In BBAT, patients focus more on bodily features, such as breathing, grounding, stability, centring, flexibility and flow [36]. The patients undergoing BBAT are not systematically trained to identify and describe feelings but they are encouraged to observe and accept sensations and emotions that awaken during the treatment sessions [36]. As neither of the two interventions is ‘placebo’, we think it is important to compare both ASSA and BBAT with treatment as usual.

We have chosen reduction of depression symptoms as the primary outcome measure, even though some patients were not depressed at the time of inclusion. A reduction in depression symptoms might be of value even for patients not fulfilling our criteria for self-reported depression. A lower cut-off level (≥5 points) has been proposed for the depression subscale of the Swedish version of Hospital Anxiety and Depression Scale than we have chosen to use. This means that the sensitivity for depression might be less than optimal in our study [39].

One limitation to our studies is that we only use self-report instruments, not structured interviews. Another limitation is that not enough patients in the first part of our randomized controlled trial agreed to participate in the intervention, so randomizing into three arms (ASSA, BBAT and a no-intervention group) was impossible. We aim to recruit more patients in Study II, to be able to perform the three-arm randomized controlled trial.

We foresee recruitment difficulties. Patients have generally not previously been introduced to the idea that emotional factors might impact their disease; presumably neither ASSA nor BBAT are methods known to the patients. Patients may feel that it is stressful to talk about emotions in a group situation and therefore BBAT may be more easily accepted.

Another difficulty is that most patients are working or university students, which might make participation difficult, therefore the Swedish Social Insurance Agency will offer preventive sick-leave compensation for treatment session time. It is, however, not possible to offer preventive sick-leave compensation for nonparticipants.

The clustering within the pair of instructors in ASSA might affect the results, but this will be tested. The three ASSA instructors were trained together, and have previously been ASSA instructors together in all three possible combinations; this will probably minimize the effects of clustering. The three BBAT instructors all have special training and long experience, but have not been trained together; however, they will co-operate in synchronizing how they will perform the BBAT intervention. The results of the interventions might be influenced by the group members being randomized into each group and by study design. Previously, we have carefully selected patients who we thought could benefit from the ASSA intervention and would not disturb the group dynamics. In the current studies, no individual selection can be made by the instructors, owing to the study design. Previously, we have made contracts with the patients to do their very best to participate in all sessions. In the current studies, we have declared that they can stop participating at any time.

In summary, depression is a serious disease in patients with diabetes. Intervention with ASSA and BBAT will be tried for patients with diabetes, psychological symptoms and inadequate glycemic control, first in one two-arm randomized controlled trial and then in one three-arm randomized controlled trial.

## Trial status

Patients from the hospital outpatient clinic were recruited in 2009 with the final follow-up section expected to take place in 2016. Recruitment from primary care centres will start in 2016.

## Abbreviations

ASSA: affect school and script analysis
BBAT: basic body awareness therapy
CONSORT: Consolidated Standards of Reporting Trials
IFCC: International Federation of Clinical Chemistry
NGSP: National Glycohemoglobin Standardization Program
